# Using COVID-19 Pandemic as a Prism: A Systematic Review of Methodological Approaches and the Quality of Empirical Studies on Physical Activity Behavior Change

**DOI:** 10.3389/fspor.2022.864468

**Published:** 2022-04-21

**Authors:** Ralf Brand, Sanaz Nosrat, Constantin Späth, Sinika Timme

**Affiliations:** ^1^Sport and Exercise Psychology, University of Potsdam, Potsdam, Germany; ^2^Department of Kinesiology, Iowa State University, Ames, IA, United States; ^3^Department of Health Sciences, Lehman College/City University of New York, New York, NY, United States

**Keywords:** meta-science, exercise, methods, quality, study designs, standards

## Abstract

**Background:**

The COVID-19 pandemic has highlighted the importance of scientific endeavors. The goal of this systematic review is to evaluate the quality of the research on physical activity (PA) behavior change and its potential to contribute to policy-making processes in the early days of COVID-19 related restrictions.

**Methods:**

We conducted a systematic review of methodological quality of current research according to PRISMA guidelines using Pubmed and Web of Science, of articles on PA behavior change that were published within 365 days after COVID-19 was declared a pandemic by the World Health Organization (WHO). Items from the JBI checklist and the AXIS tool were used for additional risk of bias assessment. Evidence mapping is used for better visualization of the main results. Conclusions about the significance of published articles are based on hypotheses on PA behavior change in the light of the COVID-19 pandemic.

**Results:**

Among the 1,903 identified articles, there were 36% opinion pieces, 53% empirical studies, and 9% reviews. Of the 332 studies included in the systematic review, 213 used self-report measures to recollect prepandemic behavior in often small convenience samples. Most focused changes in PA volume, whereas changes in PA types were rarely measured. The majority had methodological reporting flaws. Few had very large samples with objective measures using repeated measure design (pre and during the pandemic). In addition to the expected decline in PA duration, these studies show that many of those who were active prepandemic, continued to be active during the pandemic.

**Conclusions:**

Research responded quickly at the onset of the pandemic. However, most of the studies lacked robust methodology, and PA behavior change data lacked the accuracy needed to guide policy makers. To improve the field, we propose the implementation of longitudinal cohort studies by larger organizations such as WHO to ease access to data on PA behavior, and suggest those institutions set clear standards for this research. Researchers need to ensure a better fit between the measurement method and the construct being measured, and use both objective and subjective measures where appropriate to complement each other and provide a comprehensive picture of PA behavior.

## Background

On March 11, 2020 the World Health Organization (WHO) declared the new coronavirus disease (COVID-19) a pandemic (World Health Organization, 2020). Governments and nations almost everywhere in the world responded quickly by installing non-pharmaceutical interventions (NPIs) to mitigate the spread of SARS-CoV-2 (Ritchie et al., 2020). In early 2020, the five most frequently taken measures by the governments were “educate and actively communicate with the public,” “mass gathering cancellations,” “crisis management plans,” “closure of educational institutions,” and “small gathering cancellations” (Desvars-Larrive et al., 2020).

The NPIs imposed have affected many aspects of people's lives including physical activity (PA), sport, and exercise. This is problematic because it is known that PA and exercise can contribute positively to physical, social and mental health (Kramer, 2020; Posadzki et al., 2020). Insufficient PA is not a recent public health problem and has been considered as one of the great challenges of our time even under “normal” prepandemic conditions (Guthold et al., 2020). In fact, warnings that physical inactivity should be considered a pandemic (Kohl et al., 2012, p. 294) were issued by medical experts long before COVID-19.

Early in the pandemic, scientists in multiple fields were challenged to answer fundamental questions, develop testing, provide guidelines, and work toward providing vaccines. Exactly 1 year after COVID-19 was declared a pandemic, on the same day in March 2021, 2013.06 million people worldwide had at least received their first dose of COVID-19 vaccine (Mathieu et al., 2021) underlining the effectiveness and importance of these scientific efforts (Subbarao, 2021).

At the same time, scientists published their expectation that PA could potentially reduce the morbidity and mortality of viral infection with SARS-CoV-2 (da Silveira et al., 2021). However, the governmental restrictions might have encouraged physical inactivity. Behavioral epidemiology called for action suggesting an international PA and public health research agenda to inform policies and practices (Sallis et al., 2020). The question is how researchers in the field of PA, sport, and exercise responded to the urge for more information.

To provide an answer, we aim to investigate the responsiveness of the research community within the first year of the pandemic, and help evaluate their performance. In doing so, our study will not focus on synthesizing the empirical findings reported in the original publications. Rather, we will summarize and take a critical look at “apparent” standards in this area of research, and highlight how studies were conducted. This work represents an effort in contributing to meta-research (Ioannidis et al., 2015) which will help advance future research in the field of PA, sport, and exercise.

### Systematic Literature Reviews in Criticism

In general, systematic reviews aim to collate all empirical evidence from studies that meet predefined criteria and contribute to answering a specific research question (Campbell Collaboration, 2021; Higgins et al., 2021). The PRISMA statement defines reporting standards for systematic reviews to help ensure that the evidence is accurately summarized (Liberati et al., 2009; Rethlefsen et al., 2021). The overall goal of this approach is to secure, through a strictly rule-guided process, the replicability of conclusions about the state of the research, so that the best evidence is provided for policy and practice. On the other hand, narrative reviews aim to evaluate the state of research through the critical intellectual appraisal of findings by academic experts (Ferrari, 2015). In this type of review, deciding whether or not a study is relevant in answering a research question is subject to the expert's more subjective assessment of importance of the contribution. Compared with systematic reviews, narrative reviews are often but perhaps unfairly considered to be of lower quality (Greenhalgh et al., 2018).

Reviews of behavioral changes due to the COVID-19 pandemic have also been published in the field of PA, sports, and exercise. We will also include these in our analysis.

This is because, in recent years, there has been a surge in the number of criticisms on systematic reviews, perhaps due to the increased volume of publications of such reviews (MacLure, 2005; Yuan and Hunt, 2009; Ioannidis, 2016; Hammersley, 2020). For example, it was pointed out that the term “systematic” does not automatically mean “high quality,” but it obviously refers to “a set of methodologies characterized by tight focus, exhaustive search, high rejection-to-inclusion ratio and an emphasis on technical rather than interpretive synthesis methods” (Greenhalgh et al., 2018, p. 2). Some authors highlighted that systematic reviews often fail to adequately account for the complexity of real-world phenomena and influential contextual factors pointing out that they often are not truly useful for policy making (Booth et al., 2019). It was pointed out that systematic reviews are often poorly conducted (Whitty, 2015) or written in a way that is not suitable for policy decision making (Greenhalgh and Russell, 2006). It was also indicated that privileging of certain studies by authors as high-quality evidence due to their approach and method introduces a systematic bias that contradicts the central claim of systematic reviews which is to provide non-biased evidence (Boell and Cecez-Kecmanovic, 2015). In sum, at the center of the criticism is the often-mechanistic adoption of the procedure accompanied by a lack of intellectual contribution (Boell and Cecez-Kecmanovic, 2010; Greenhalgh et al., 2018).

Therefore, to avoid some of these pitfalls, our systematic review was guided by one extra consideration: According to Boell and Cecez-Kecmanovic (2014), systematic reviews lose value and meaning if the highly structured approach to searching and acquiring original studies (steps in this cycle are “searching,” “sorting,” “selecting,” “acquiring,” “reading,” “identifying,” “refining,” and eventually back to “searching”) is at the expense of intellectual analysis and interpretation of those studies. They emphasized that an additional hermeneutic cycle is needed to substantiate the steps of “identifying” and “refining” by adding the steps of “mapping and classifying” and “critical assessment” of the received information, so that the “argument development” and iterative reframing of the “research problem/question” may benefit.

For our study, this means that while we will strictly adhere to the guidelines for conducting and reporting a systematic review (Siddaway et al., 2019), we will begin our analysis with explicating the context and hypothesizing about how researchers would likely design their research questions and studies. In the field of research synthesis, this is proposed as a means to help conceptualize complex systems by transparent reflection on initial assumptions about the research question. This approach helps, for example, to make underlying assumptions about causal relationships more explicit in order to avoid mechanical integration of studies without analysis and reflection (Anderson et al., 2011; Petticrew et al., 2019).

### This Review

Shortly after COVID-19 was declared a pandemic (on March 11, 2020) and restrictions were put in place, a once-in-a-lifetime opportunity for research in PA, sport, and exercise presented itself to explore the behavioral changes made specifically to adapt to these early restrictions. For example, 4 weeks after March 11, in 57 countries, individual sectors were closed including commercial and public facilities for sports, fitness and physical education (Hale et al., 2021). Behavioral researchers would probably never get a similar opportunity to study the effects of such a serious intervention for the first time.

We expect that the researchers' response to the pandemic was rapid and that—given the prominence of the problem—many colleagues worldwide contributed quickly to this research. Further expectations are that, to accurately describe and more closely analyze changes in behavior that occurred during the initial lockdown, data should have been collected at two time points of pre and during the pandemic. To control for cohort effects, within-subject repeated measures would be considered ideal compared to other designs. For measurement of PA, both self-report and device-based options are available each with its own pros and cons. Self-report measures are often easily accessible and practical, while prone to reporting bias and poor memory recall; device-based measures are more expensive and cumbersome to use, while being more accurate (Nigg et al., 2020). For example, higher measurement reliability is achieved when “intensity,” “frequency,” and “duration” are measured objectively *via* accelerometry and locomotion *via* GPS, whereas the “type” of PA is probably still best measured through self-report. Therefore, we expect that researchers have made specific use of both options. In addition, when the goal is to accurately describe the behavior of a population based on the sample, representative or random samples are superior to *ad-hoc* samples. Accordingly, we hope to find at least some studies with results that can be generalized to broader populations. In sum, we expect the research conducted either in a way to guide policy makers in their decisions or to increase basic knowledge and/or test theories on PA, sport, and exercise.

With regard to pandemic-related changes in PA, sport and exercise behavior, it would be naive to assume that the aforementioned restrictions and containment measures had no impact on PA, sport, and exercise behavior. For example, for those who were not used to solo exercise, or those who needed facilities for their preferred type of exercise, the restrictions meant an imminent need to change their habits. Therefore, even if a general decline in PA, sport, and exercise would have been the most plausible (albeit trivial) hypothesis for empirical studies, we expected that changes in PA, sport and exercise would be analyzed more closely in terms of time/duration, frequency, intensity, and type. Moreover, all these variables could have been affected differently during the initial lockdown compared to months after.

More specific assessments like these are particularly useful when analyzing the different physiological (e.g., changes in cardiovascular fitness or muscular strength) and psychological (e.g., changes in well-being and mood) effects. In addition, these assessments could provide useful information for more nuanced policy making (e.g., impact of school closures and cancellation of physical education on child motor development).

To address what we consider appropriate criticism of the often too mechanistic approach in many systematic reviews, we will follow PRISMA guidelines, but take Boell's and Cecez-Kecmanovic's (Boell and Cecez-Kecmanovic, 2015) recommendations for intellectual substantiation and put extra effort into describing, classifying, and critically assessing the totality of search results. Thus, the systematic review will be preceded by a publication analysis and thematic mapping of all retrieved articles. This may allow an even more in-depth assessment of the state of development of the research field.

## Methods

### Protocol

Our protocol was prepared according to the PRISMA-P statement (Moher et al., 2015). The final version of the protocol is given in Supplementary Table 1. This protocol is not registered.

### Eligibility Criteria

We included any reports (including opinion pieces, as well as empirical studies) related to COVID-19, PA, sport, and exercise behavior. They had to be published within the 365 days (1 year) after WHO declared COVID-19 a pandemic and had to be in English or German. Those that did not primarily address human subjects were excluded. We included published articles on PA, sport, and exercise according to the following definitions. “Physical activity” is any bodily movement generated by skeletal muscles involving energy expenditure. “Sport” is defined as a PA performed in the context of competition and contest. According to a standard definition “exercise” is referred to as a subset of PA that is planned, structured, and repetitive and has as a final or an intermediate objective of improvement or maintenance of physical fitness (Caspersen et al., 1985).

### Information Sources and Search Strategy

To identify potentially relevant documents, a comprehensive literature search was conducted by the authors on February 28, July 15, and July 16, 2021. We searched the Social Sciences Citations Index, the Emerging Sources Citation Index, Medline, PubMed Central (PMC) and all other sources included in the Web of Science platform and the Pubmed database.

The search string used was [(“Physical activit^*^” OR “Exercis^*^” OR “Sport^*^”) AND (“COVID^*^” OR “SARS-CoV-19”)]. All searches were restricted by the filters “Human,” “English,” and “German”. The other filter used was date restrictions. The search in February was set from “January 1, 2019 to February 27, 2021,” and the search conducted in July was set from “February 27, 2021 to March 11, 2021”.

### Article Selection Process

Search results were imported and processed in the Covidence systematic review management online system. The reviewer team consisted of three experienced researchers (RB, ST, SN) and five graduate students (CS, and four others), each with different roles and qualifications in the project. Team training exercises were conducted prior to each step of the screening and article selection processes.

In the initial title and abstract screening, two independent reviewers marked for inclusion any title/abstract that was determined to fall into the category of empirical research, review article, or meta-analysis providing direct evidence of PA, sport, or exercise behavior change. Eligibility of these studies was again verified in a full text screening by two independent reviewers, before it was released for data extraction for the systematic review. Disagreements were always resolved through discussion between the two reviewers. Of the two reviewers, one was always from the group of the experienced researchers.

The records that did not contain behavior change data but addressed PA, sport, and exercise differently were included in an extra analysis to reflect the thematic range of all articles found (articles not focusing on human behavior or the social or cultural significance of sport and exercise were excluded at this stage). This process was conducted outside of Covidence using spreadsheets.

### Data Extraction

For all articles, the standard bibliographic information (e.g., title and abstract of the article, year of publication, and journal title), the nationality of the first author *via* his professional affiliation, and the exact publication date were extracted. In addition, for items included in the systematic review on behavior change, methodologically relevant information was extracted. These included characteristics of the sample (e.g., nationality and study population), study design (e.g., duration and type of sampling relative to the stringency of the health containment measures in the respective country, where possible; number of measurement time points), measurement method (e.g., device-based measurement, self-report), and measurement content (e.g., names of questionnaires used). A complete listing of all extracted information is part of the Supplementary Table 2 to this review.

The data extraction sheets were tested for accuracy with 20 randomly selected articles and were modified as needed based on the team feedback. Each included document was extracted by one team member and verified by a second reviewer from the group of experienced researchers (RB, ST, SN).

### Methodological Quality and the Risk of Bias

For the systematic review section, following recommendations (Ma et al., 2020), standardized risk of bias assessment was based on criteria from the JBI checklist for analytical cross-sectional and prevalence studies (Moola et al., 2020), and the AXIS appraisal tool for cross-sectional studies (Downes et al., 2016). In addition, extracted data were used for the appraisal of methodological quality as detailed in section “Overview of the Methodologies”.

### Synthesis of the Results

A machine learning technique that is topic modeling with Latent Dirichlet Allocation analysis (Grün and Hornik, 2011) was used to describe the thematic scope of all published articles *via* their article titles. The stringency of confinement measures during data collection were taken from the Oxford COVID-19 policy response tracker for each country (Hale et al., 2021). Statistical analyses were conducted with the R programming environment (R Core Team, 2021). Evidence mapping was used to highlight some of the most important findings (Miake-Lye et al., 2016).

## Results

### Literature Search

**Figure 1:**
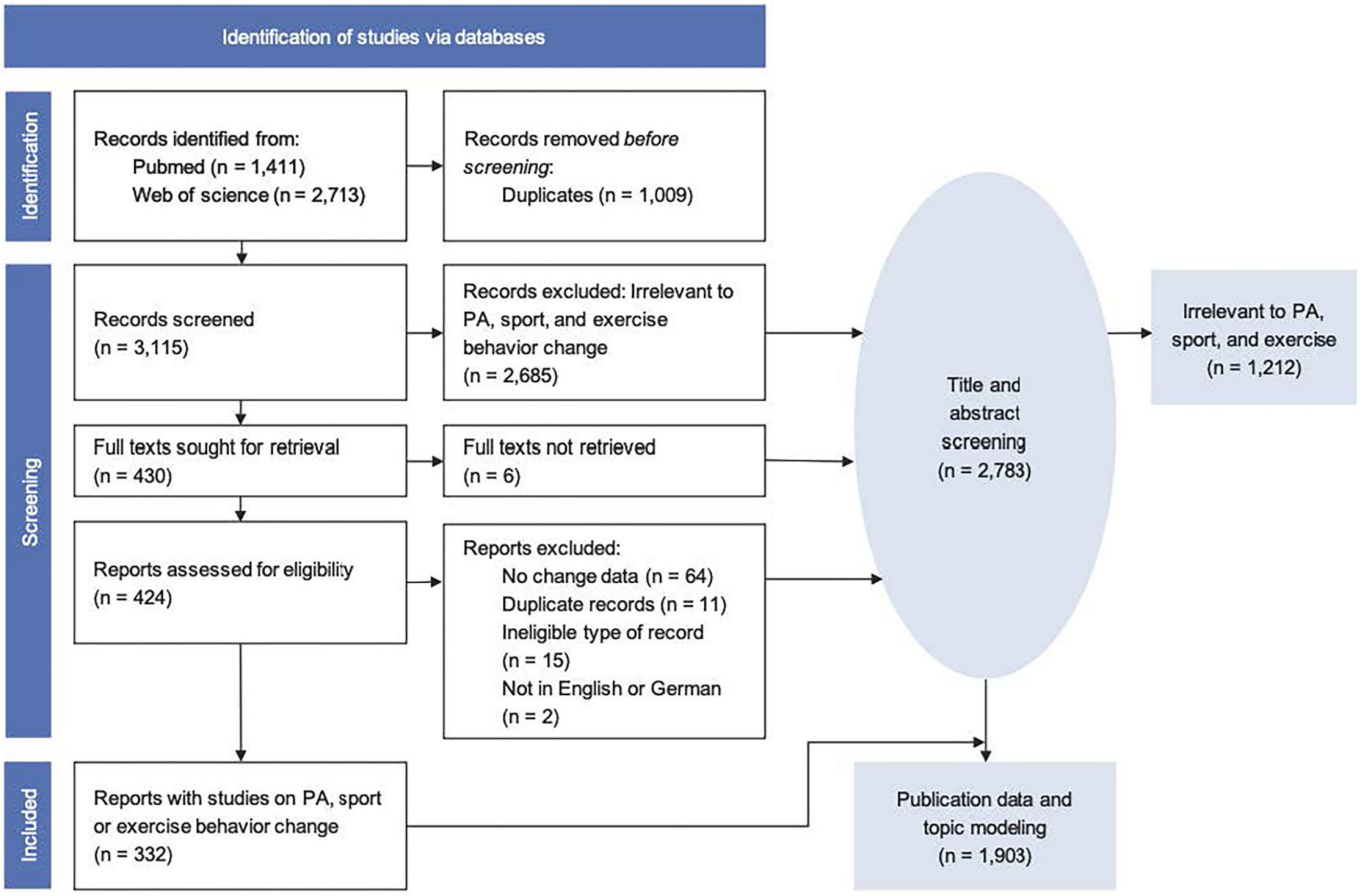
Study flow.

The literature search resulted in 4,124 citations. After removal of duplicates, 3,115 documents were screened by title and abstract and we excluded any articles that did not discuss or analyze consequences of the COVID-19 pandemic for PA, sport, or exercise behavior. A total of 1,903 documents were included for the analysis of publication data (i.e., number of published articles from each country, publication dates, and types) and the illustration of the thematic range in all articles. For the systematic review, after removal of articles with no data on PA behavior change, a full-text screening was performed for 430 articles and 332 were finally included. Reasons for exclusions are listed in the flowchart (Figure 1; full citations listed in Supplementary Table 2).

### Thematic Analysis (*n* = 1,903)

**Figure 2:**
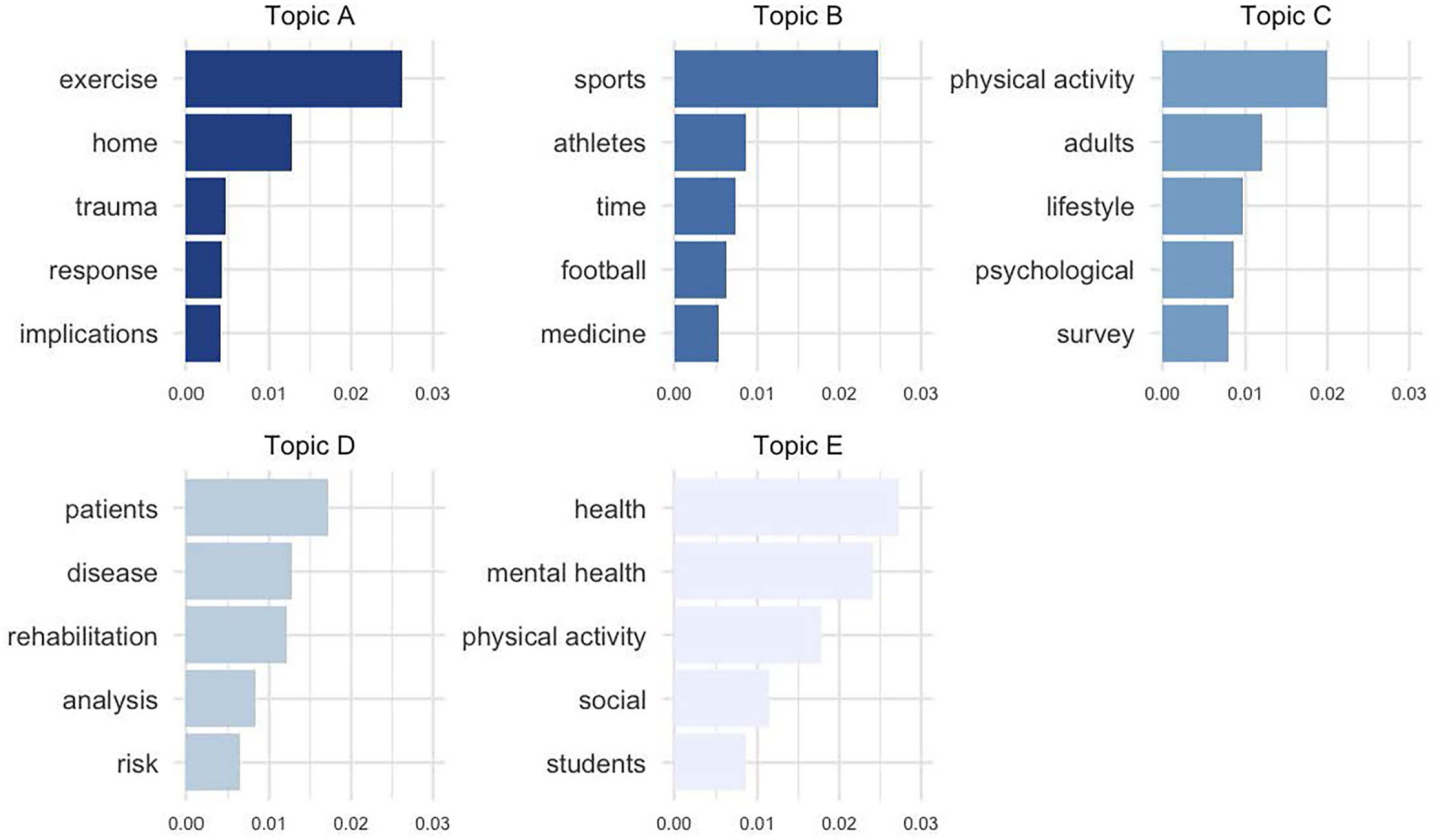
Topic analysis of articles related to PA, sport, and exercise. Results of the statistical topic analysis conducted on the titles of the 1,903 identified articles that have been published in the first 365 days of COVID-19 pandemic as declared by WHO. The analysis suggested five topics, each delineated by the five words/terms shown. Numerical values on x-axis are beta values which are the probability of a term/word generated from that topic as computed by the model.

Topic analysis suggested five fields of interest as reflected in the titles of all identified published articles. The five terms (words and word pairs) that primarily characterize the five topics according to their beta values are illustrated in Figure 2. The topics are: A. focus on exercise and home confinement (topic prominence, i.e., the number of unique documents where a topic appears, *n* = 756); B. focus on sports and athletes' return to play (topic prominence: *n* = 807); C. focus on PA and psychological outcomes (*n* = 825); D. focus on patient population and rehabilitation (*n* = 765); E. focus on subjective well-being and PA (*n* = 820). Taken together, these numbers indicate that issues related to preventive health care were addressed approximately three times more frequently (topics A, C, and E) than those related to sports and athletes (topic B) and those related to patients and rehabilitation (topic D).

**Figure 3:**
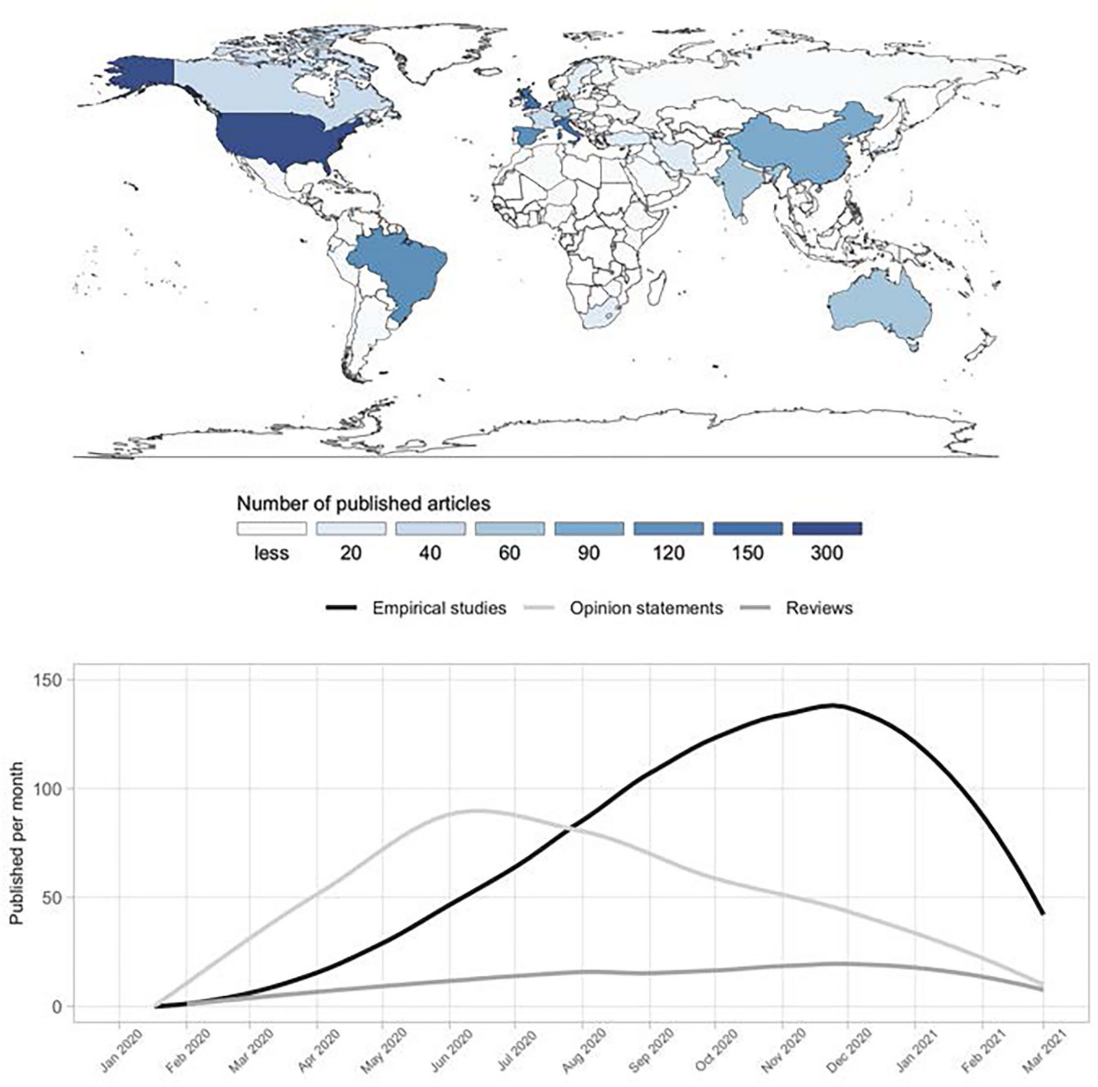
Timing of publications and contributions from countries. Number of published articles per month (top), and publication types and dates of all articles (*N* = 1,903) published on PA, sport, and exercise in the first 365 days of COVID-19 pandemic as declared by WHO (bottom).

Figure 3 shows the trend in the number and type of publications within the first 365 days of COVID-19 pandemic. A total of 1,005 empirical studies (representing 53% of the identified 1,903 articles), 694 opinion pieces (36%), 169 reviews (9%), and 35 case reports (2%) were published during this time. Opinion pieces on varied topics dominated between April and July 2020 with an average of 3 per day (almost 80 per month). From around August onwards, the number of empirical studies prevailed, with 137 published in December alone. From July to December 2020, almost 2 reviews were published per day, mostly narrative reviews without any in-depth analysis. Finally, there was a steep decline in the numbers of all publication types from January to February 2021.

Researchers from almost all regions of the world contributed to this body of literature (Figure 3). Most publications were from the USA (*n* = 348), the UK (*n* = 182), Italy (*n* = 168), Spain (*n* = 133), Brazil (*n* = 126), China (*n* = 124), Germany (*n* = 73), Australia (*n* = 72), India (*n* = 67), and Canada (*n* = 55). The proportion of opinion pieces was highest in the USA and Brazil (49 and 50%) and lowest in Spain and China (20 and 17%).

### Empirical Studies With Data on Behavior Change (*n* = 332)

Of the 1,903 articles identified, 332 (17.4%) measured data that allow to make conclusions about potential changes in PA, sport, or exercise behavior. All of the 332 studies, including the study characteristics discussed below, are listed in an Excel spreadsheet in Supplementary Table 2 which can be navigated *via* sort and filter function. In order not to inflate the reference list of this article, citations are provided only for the studies we have highlighted to discuss due to their methodological advantages.

#### Responsiveness of the Research Field

Among the countries with publications on PA behavior change, the USA (*n* = 43), Italy (*n* = 35), Spain (*n* = 35), and UK (*n* = 30) contributed most, followed by Brazil (*n* = 19), Germany (*n* = 17), China (*n* = 14), Australia (*n* = 13), Canada (*n* = 13), and France (*n* = 11) (This is an identical list to the top 10 countries with the largest number of published articles listed above, except that France appears instead of India in this list).

**Figure 4:**
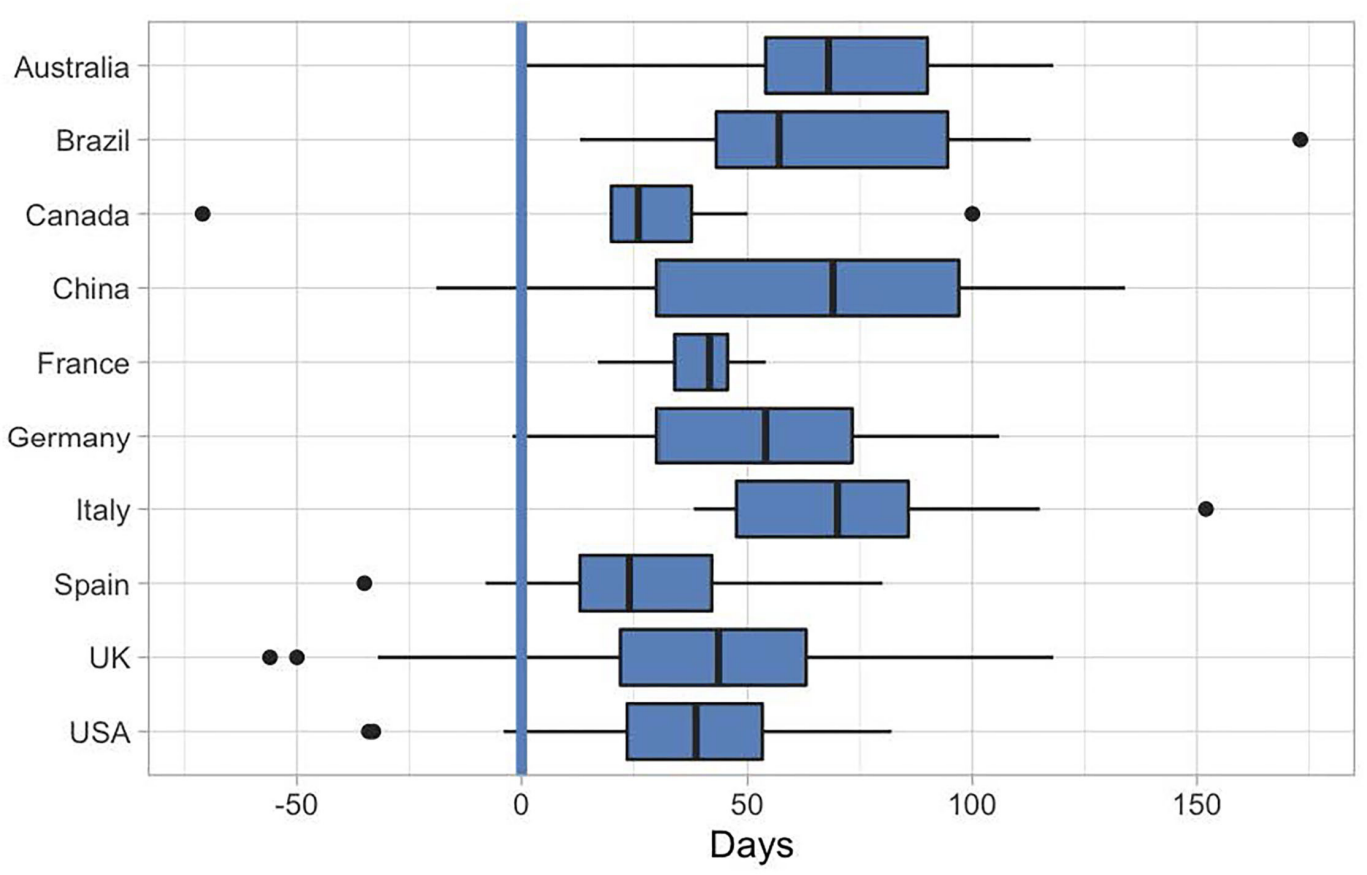
Timing of participant sampling in relation to pandemic development. The speed with which researchers began collecting data on PA behavior change in the top 10 countries that contributed with the greatest number of studies. Day 0 is the day when the most severe confinement measures were installed in the respective country (according to the Oxford COVID-19 Government Response tracker; Hale et al., 2021).

The speed with which researchers began to collect data from the first day of the strictest confinement measures in their countries (confinement index > 20 according to the Oxford COVID-19 Government Response tracker; Hale et al., 2021) was 44 days (median value). Authors from Canada, the UK, Spain, and the USA were the first to collect data in this field. This data is shown in Figure 4.

There are 272 studies with exact information on sampling time. Among these, 21.7% collected data during the period when the containment measures reached maximum strength in their countries; while 45.2% of the studies concluded their data collection before, and 33.2% of the studies started it after these containment measures reached maximum strength in their countries.

#### Overview of the Methodologies

Among the 332 studies, there were 27 studies that compared the incidences of sport injuries treated in medical hospitals before and during the pandemic. These articles show that the number of sports or exercise injuries decreased by 76% during the initial lockdown in 2020. Although, decrease in the incidents of injuries is an indirect implication of changes in exercise and sports behavior, these studies provide relatively robust data representative of the region in which medical hospitals are located.

**Figure 5:**
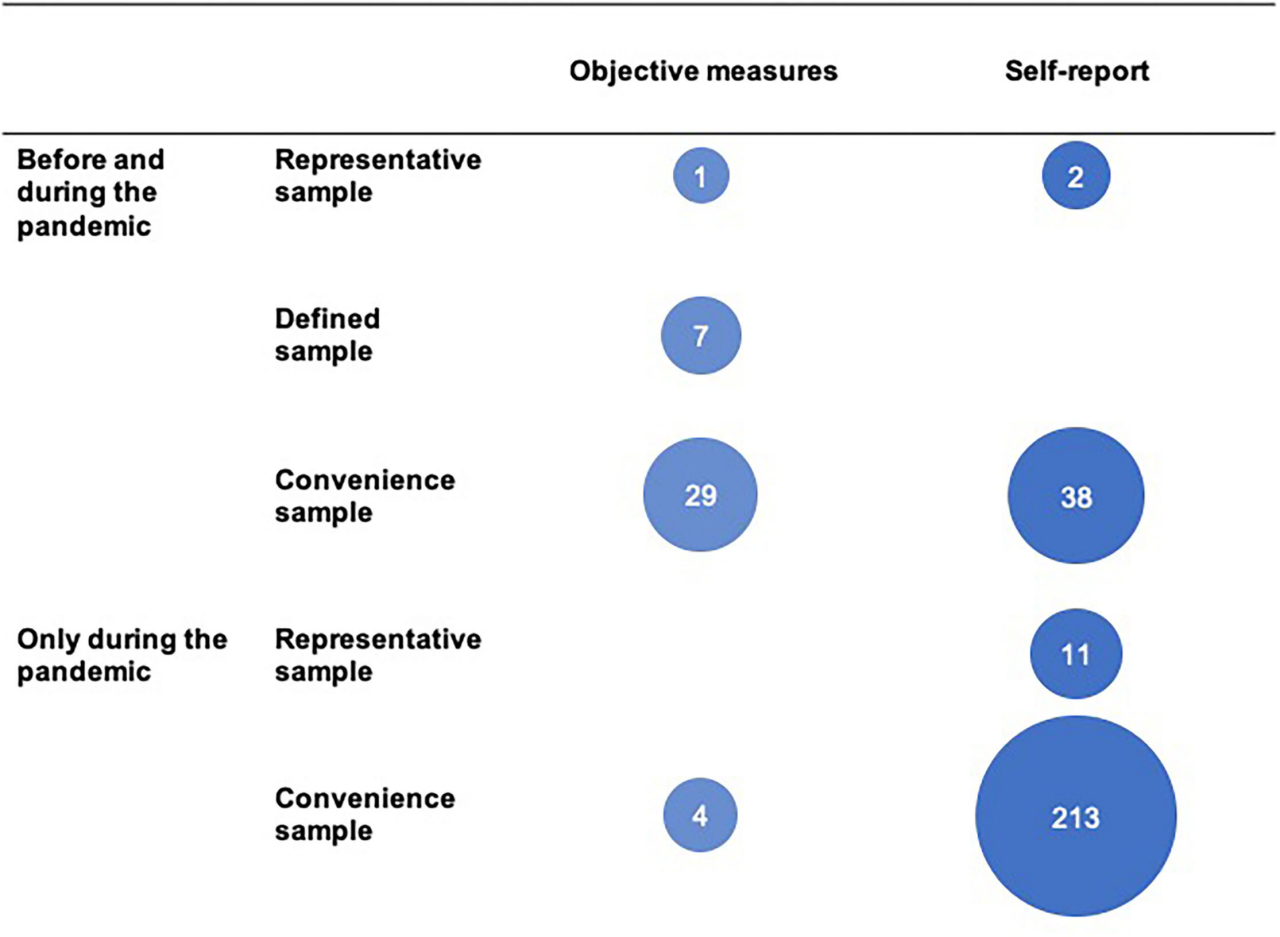
Empirical approaches of the published articles (*n* = 305; injury studies not included).

Since these articles did not collect direct measure of changes in exercise and sports behavior, we will not further include them in the discussion below and therefore we will refer to a total of 305 studies when giving proportional data in the following sections. Among these 305 studies, there were significant differences in study design and data collection (Figure 5).

##### Studies With Self-Report Measures

Of the 305 studies on PA, sport, or exercise behavior change, 264 used questionnaires. Less than 5% of these studies collected data on change in exercise type (e.g., García-Tascón et al., 2020; Schnitzer et al., 2020) or focused on whether cardiovascular endurance exercise was affected differently than muscle strengthening exercises (e.g., Leiros-Rodriguez et al., 2020; Zaworski et al., 2020; Naughton et al., 2021). In 96 studies (36.4%) participants were only asked whether they were currently doing “more,” “less,” or “about the same” amount of PA during the pandemic compared to before the pandemic. A smaller number of studies quantified behavior change in greater detail (23.1%; *n* = 61), for example collecting data on PA frequency, PA minutes per week, or MET (metabolic equivalent) minutes per week. International Physical Activity Questionnaire (IPAQ) was frequently used by researchers (in 54 studies). This questionnaire can be used to inquire the number of days per week (usually during the last 7 days) and the number of minutes and hours per day of moderate- and high-intensity PA (plus time spent walking and sitting). The remaining 107 articles used a wide variety of ways to measure PA behavior change (e.g., alternative standardized questionnaires such as the Godin Physical Activity Questionnaire, one-item measures, Likert-type scale items), therefore, it is difficult to summarize, compare, and contrast their results.

##### Studies With Objective Measures

In 41 studies, researchers used objective measures as follows: 33 used measurement devices such as hip- or wrist-worn accelerometers, or apps collecting data from the participant's smartphone. Seven studies analyzed “big data” from servers such as data from fitness app users, or Google mobility data. One distinctive feature of these studies was that individuals providing data for these studies were not specifically aware of the time their behavior was being recorded for further analysis. Finally, in one study, researchers used fitness test scores to derive conclusions about potential changes in PA behavior.

##### Measurement Designs and Sampling

One in four behavior change studies (77 of the 305 studies), measured PA, sport, or exercise behavior before and during the pandemic. In nearly half (*n* = 37), behavior was directly observed with measurement devices including one study where researchers compared fitness test results from before to during the pandemic. Most studies (73.4%; *n* = 224) assessed current behavior during the pandemic and had participants recall their prepandemic behavior.

Less than 5% of the 305 studies (*n* = 14) claimed to have representative findings (i.e., a country, a region, or a specific city). More than 90% of all studies (*n* = 284) used *ad-hoc* or convenience samples. The remaining 2% of studies (*n* = 7) sourced data from narrowly defined samples such as participants in a PA intervention program or users of one particular wearable activity tracker.

#### Populations Studied, and Important Findings

**Figure 6:**
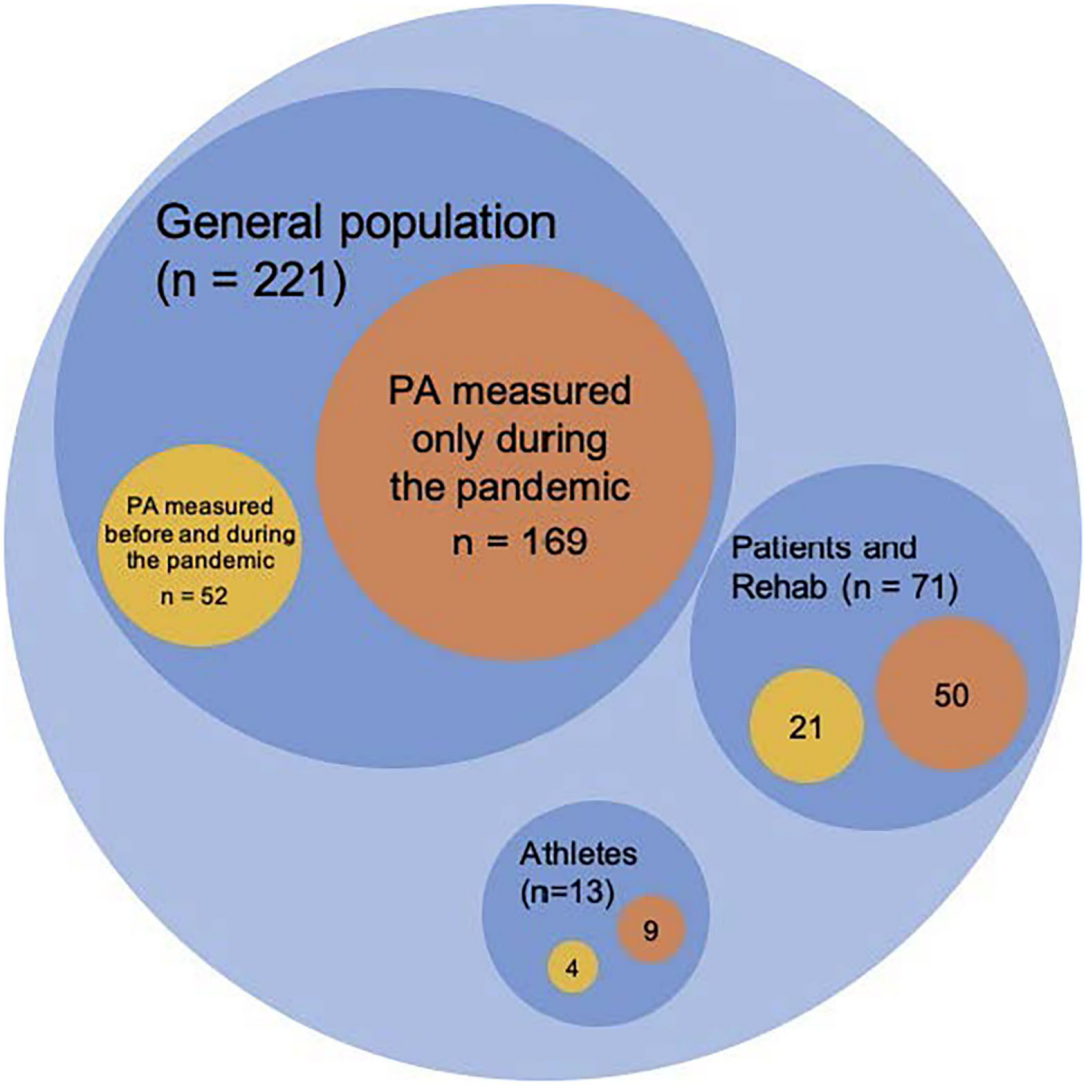
Study designs and target groups. Number of empirical articles on PA behavior change in different populations and timing of data collection (graphical proportions are not exact for readability purposes).

Below, we will categorize the total of 305 studies into 3 groups based on the population studied. In each group, we will also elaborate on the methodologies and provide a summary of the results of the studies that we consider to be of higher quality. Figure 6 provides an overview of the relative frequencies of studies that measured data at two time points, both before and during the pandemic.

##### Athletes or Regular Exercisers (n = 13)

Very few studies (4.3%, i.e., 13 out of 305) focus on participant samples with athletes or regular exercisers. All of them used convenience sampling. Below we will discuss those studies with large sample sizes to be able to generalize the results to the subpopulation of athletes and exercises studied.

There are six studies with large sample size (more than 1,000) on the subpopulation of endurance exercisers and athletes. Two of these used objective measures and compared data from before to during the pandemic (Venter et al., 2020; Scheer et al., 2021). One of these two compared competition statistics of endurance running events before and during the pandemic (e.g., number of events, number of competitors, finishing times) (Scheer et al., 2021). This study found the numbers of endurance events, and the number of finishers in these events decreased, and marathon finishing times increased during the pandemic. The second study in this group analyzed training and exercise data from thousands of users of a fitness training app for runners and cyclists, and compared the exercise volume before and during the pandemic (Venter et al., 2020). This study was conducted in the Norwegian capital Oslo and demonstrated that exercisers (“recreationists,” according to the authors) had shifted their exercise activities to city parks and places with higher outdoor greeneries. This study shows that outdoor recreational activity over Oslo municipality increased by 291% during the pandemic relative to a 3-year average for the same days before the pandemic. This increase was significantly greater than expected after adjusting for the prevailing weather and the time of year.

Three other studies were survey studies that had participants recall their prepandemic exercise routines and compared it to their routines during the pandemic; only one collected participant's responses at two time points (i.e., before and during the pandemic) (Cloosterman et al., 2021). The result of this study shows that 93.9% of runners continued running during the pandemic with mean weekly running frequency, duration, distance, and speed very similar to the prepandemic.

In sum, these higher quality studies suggest that although performance (e.g., race finish times) declined among competitive runners and cyclists during the pandemic, the total volume of running and cycling was not affected by the pandemic perhaps due to accessibility of this type of exercise despite the restrictions. Some of these studies also show that those who were already active prepandemic might have increased their activity levels during it.

##### Patients and Rehabilitation (n = 71)

One out of five studies (23.3%, 71 of the 305 identified) referred to patients or the context of disease or rehabilitation. Most of these (74.6%; i.e., 53 out of 71) were conducted with adult samples (56.4 ±14.6 years old). Nine (12.7%) were conducted with children or adolescents, and 9 (12.7%) addressed seniors. Patients or rehabilitants with diabetes-related conditions (*n* = 16) and patients with cardiovascular system impairment (*n* = 14) were frequently studied. No other groups of patients were examined with similar frequency. All studies used convenience sampling except for one cross-sectional survey study of 195 randomly selected elderly with coronary symptoms.

In about one third of the studies (28.2%; 20 of the 71 studies), researchers had data from two measurement time points, that is one before and the other during the pandemic: Twelve of these used device-based measures for PA assessment (e.g., cardiac implantable electronic devices or wrist- or belt-worn accelerometers), but only four had more than 100 participants. The remaining eight studies with repeated-measures designs relied on survey data, including one with 1,433 patients with coronary vascular disease, and one with 248 post kidney transplantation patients (all others had less than 100 participants each).

Against the backdrop of these published evidence, we believe that any generalized conclusions about changes in PA or exercise in patients and rehabilitants (as a whole) may not be valid. However, there are 16 studies with patients with coronary symptoms: In one cross-sectional survey study with the random sample of 195 elderly patients with coronary symptoms, 45.1% reported more than 25% decrease in PA, but 46.7% reported no change (Cransac-Miet et al., 2021). In the relatively large sample of 1,433 elderly patients with coronary vascular disease who were surveyed before and during the pandemic, an overall increase in moderate-to-vigorous PA (MVPA) was reported due to an increase in leisure time walking (van Bakel et al., 2021). In contrast, there are seven studies (relying on device-based measurement before and during the pandemic with smaller *ad-hoc* samples of patients with both severe and moderate heart disease) that indicate overall decrease in PA levels (Al Fagih et al., 2020; Browne et al., 2020, 2021; Malanchini et al., 2020; Sassone et al., 2020; Vetrovsky et al., 2020; Bertagnin et al., 2021). Thus, even for groups with similar clinical diagnoses, results are heterogeneous, and even more so when different pathologies are considered.

##### General Population (n = 221)

Two thirds of the studies with data on PA, sport, or exercise behavior change (66.6%; 221 of the 305 identified) addressed the general population. Some of these (*n* = 35) focused on children and adolescents. Very few (*n* = 12) addressed PA behavior change in healthy elderly. As in the sections above, we will again highlight only those showing superior methodological characteristics in order to have better understanding of changes in PA behavior.

Regarding children and adolescents, there are two studies (both based on the same data set) that collected behavior change survey data from a German representative sample before and during the pandemic (Schmidt et al., 2020; Wunsch et al., 2021). These studies show that PA levels before the pandemic correlated significantly with PA levels during the pandemic independent of gender and age groups. Although the amount of time spent in sport and exercise decreased, there was an increase in the amount of time spent in informal PA that resulted in an increase in total amount of leisure time PA pre to during the pandemic. All other studies on children and adolescents are methodologically less rigorous compared to the studies presented here.

Two other studies referring to the general population with no age differentiation are highlighted here because they investigated behavior change using (a) very large samples (b) objective measures, and (c) repeated measures (i.e., two time points). As pointed out before, individuals in these analyses were likely unaware that their behavior data were being used in a study, making reactive behavior improbable. Of these two studies, one had 742,000 participants (Pépin et al., 2020), and the other more than 455,000 participants (Tison et al., 2020). Based on their sample size, both can achieve representativeness for their defined subpopulations. These studies highlight large differences between countries. For example, while the measured number of steps per day in countries with moderate forms of lockdown decreased slightly (<10% reduction), the decline in countries with more severe forms of lockdown ranged from 25 to 54% (Pépin et al., 2020).

Other similar design studies (with more than 1,000 participants) also demonstrate reduction in time spent in MVPA. For example, in a Canadian sample, MVPA declined from 194 min per week prepandemic to 177 during the pandemic (Di Sebastiano et al., 2020). Interestingly, some of these studies also indicate that the initial decline in PA bounced back toward baseline values after several weeks/months of lockdown (Di Sebastiano et al., 2020; Pépin et al., 2020; To et al., 2021). Importantly, all the studies relying on device-based measurements (including the two highlighted studies in the paragraph above with very large samples) were conducted with comparatively physically active individuals (e.g., owners of fitness trackers).

Finally, there are eight studies reporting results from self-report questionnaires for which their authors claim representative sampling of adults or the elderly population. However, all of them surveyed participants only once during the pandemic and required them to recall their prepandemic activity levels. All these studies suggest that PA decreased during the pandemic. In some studies, decrease in PA was small (e.g., 5–10% reduction for MVPA, walking and total PA among community-dwelling elderly in Japan; Sasaki et al., 2021), while in some it was larger (e.g., from an average of 201 MVPA minutes per week to 155 MVPA minutes per week in Canada; Rhodes et al., 2020). Two of these studies highlighted that mainly participation in organized sports declined, while outdoor/indoor solo activities were not affected (Mutz and Gerke, 2020; Spence et al., 2020). Only one study focused on changes in exercise type (Schnitzer et al., 2020). These authors reported that the inhabitants of an Austrian alpine region were mainly engaged in outdoor sports such as cycling, walking, hiking, jogging or mountain biking before the pandemic. During the pandemic, many switched to walking or some form of home exercise (e.g., fitness exercises, aerobics/flexibility). According to the same study, these home-based workouts declined when the strict restrictions were lifted indicating a bounce back to the prepandemic exercise routines.

It is important to point out that the way the data is reported in these studies might affect our perception of change in PA behavior. For example, some authors reported their data emphasizing the proportion of individuals who maintained their PA such as the study from Canada which reported that 58% of the participants maintained their prepandemic exercise behavior (Spence et al., 2020). On the other hand, other authors reported their data emphasizing the proportions of individuals who decreased their PA such as the study from Germany that emphasized that 31% scaled down their leisure time sport and exercise activities (Mutz, 2020). In addition, we believe it is important to put the reduction in PA time into perspective. Several studies showed a significant reduction in PA volume. However, given the current WHO recommendation that adults (aged 18–64 years) should accumulate at least 150–300 MVPA minutes per week (and perform muscular strengthening activities including all major muscle groups at least twice a week, as well as reduce sedentary time) this reduction in PA time probably did not represent a particular health risk as the total volume was still within the recommended range (Bull et al., 2020).

#### Standardized Assessment of Methodological Quality

**Figure 7:**
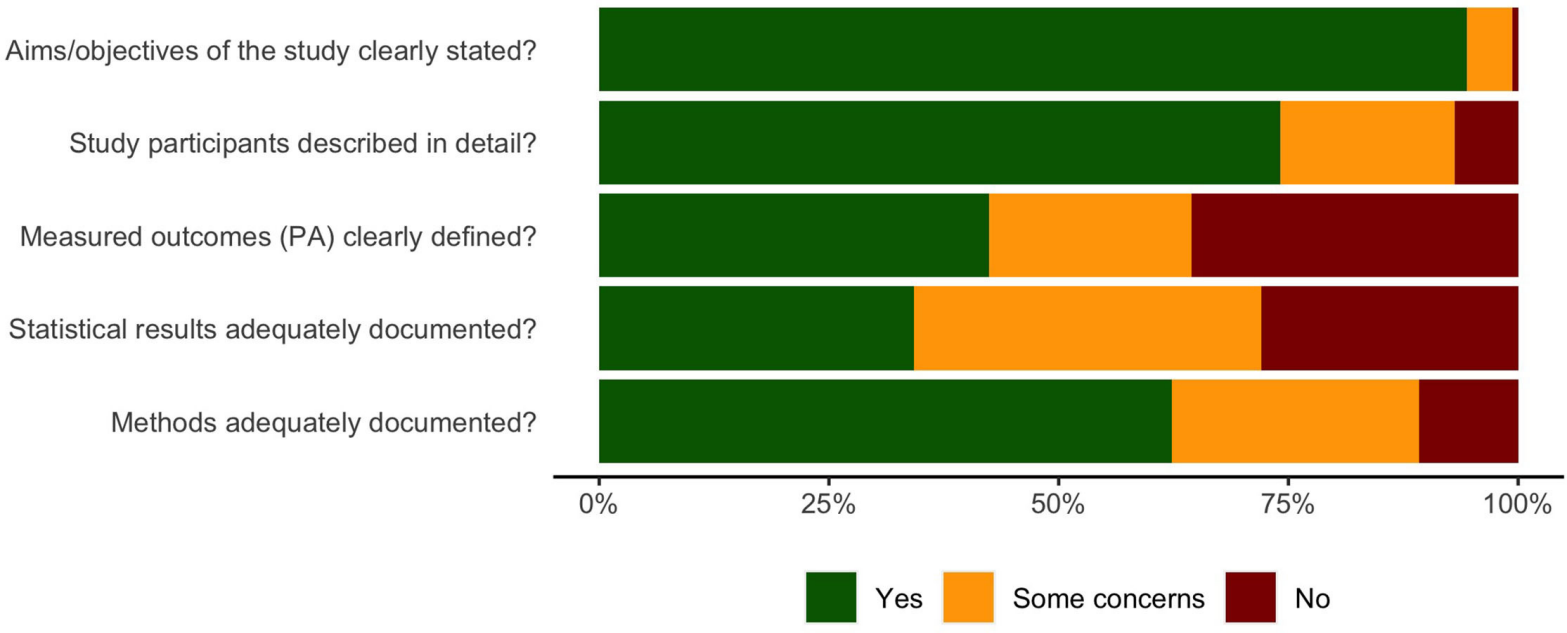
Standardized assessment of methodological quality.

Figure 7 summarizes the results of the standardized assessment of the methodological study quality using two standardized instruments. The result of this evaluation shows that many studies (*n* = 305 studies on PA behavior change) are lacking quality in two main domains. One is the clear description and differentiation of PA measured. For example, in many instances (36%), it is not clear whether the authors are reporting on structured/planned PA (e.g., going for a run, doing calisthenics) or less structured PA (e.g., short walk to a store, etc.). Second, close to one third of studies (28%) lack sufficient reporting of key statistical analysis such as statistical coefficients or test results.

### The Status of Early Published Reviews

Within the first 365 days after WHO declared COVID-19 a pandemic, four reviews were published that addressed and evaluated empirical data on changes in PA, sport, and exercise.

First, one scoping review (Caputo and Reichert, 2020) was published November 3 (7 months and 24 days after March 11, 2020) that aimed to identify the available evidence related to PA and the initial COVID-19 outbreak. The authors aimed to include all evidence from studies with any type of objectively or subjectively measured PA data. They found 41 articles published between January and July 23 (4 months and 13 days after March 11, 2020) and concluded that there was a decrease in the frequency and duration of PA, regardless of how it was measured. Slightly more than half of the articles (*n* = 23) in this scoping review (Caputo and Reichert, 2020) were also included in our systematic review, but our search also yielded 114 additional studies unidentified by this review. The authors of this scoping review did not quantify how precisely PA decreased (but emphasized that some of the reported changes were expected given the pandemic-related restrictions), did not differentiate on how specific aspects of PA, sport, and exercise were affected (e.g., whether individuals switched from muscle strengthening and general fitness activities to running), did not provide any information on the methodological qualities and validity of the results of the studies included, and did not provide information on different stringencies of confinement measures in place in different countries.

On December 15, a systematic review (Violant-Holz et al., 2020) was published focusing on the articles reporting empirical findings on psychological health and correlation with PA levels. The authors included 28 articles published by July 15. They concluded an increase in sedentary behavior and a decrease in PA levels among adults during lockdown, leading to diverse psychological outcomes. The authors did not quantify the decrease in PA (perhaps because the focus of the systematic review was psychological outcomes). They did not evaluate the validity of the results of the studies included in their review either.

There was another systematic review (López-Valenciano et al., 2021), published by January 15, 2021, focusing on the impact of the pandemic on university students' PA levels. It included 10 articles published before October 20, 2020. The authors concluded that walking, moderate, vigorous, and total PA levels decreased. They also indicated that those who met the PA guidelines before the pandemic, stayed active and still met the PA guidelines during the pandemic. All studies included in this review were also identified and included in our systematic review. We identified 7 additional studies published during this period (before October 20, 2020). The authors commented on the methodological quality of the identified studies and stated that they included 5 with a high risk of bias. There is no indication in the article whether accounting for methodological quality could have affected the authors' overall evaluation and conclusions.

Finally, another systematic review (Stockwell et al., 2021) was published on February 1, 2021 with the goal of synthesizing published evidence on PA and sedentary behavior change. The reviewers included 66 articles published by October 2020 (no specific date given) that investigated change in any form of PA before and during the pandemic. Their key finding is that during the pandemic the overall PA among healthy adults and children decreased compared to prepandemic. The authors particularly highlighted that there were three studies showing those who were more active prepandemic were more likely to show larger decrease in PA during it. They also mention reductions in PA for participants with medical conditions, while excluding those with eating disorder. For elite athletes, large decreases in both training volume and training intensity and corresponding decrease in post-lockdown physical performance tests were reported. The authors refrain from quantifying the overall trend of observed decrease in PA, or changes in PA or exercise type. Effects of contingency measures of varying stringency are not discussed. A total of 48 studies that were identified by this earlier systematic review (Stockwell et al., 2021) were also identified and included in our systematic review. Our own search yielded 110 additional articles for the same period that were not included in in the earlier one. The authors reported detailed quality assessment of included studies. However, there is no indication of whether this variable had any role in summarizing and evaluating the overall results. One distinctive (yet not necessarily advantageous) feature of this systematic review was that the authors excluded all studies for which no institutional ethical approval was reported.

Overall, these earlier reviews have heterogeneous findings, and show lack of in-depth analysis of study results and their validity. Additionally, the majority of these reviews did not differentiate between the results of methodologically robust studies vs. those of weaker designs. More importantly, many of these reviews made definitive conclusions about decrease in PA based on the studies they categorized as low quality. In our opinion, it is hardly possible that accurate conclusions on changes in PA behavior could be drawn on the basis of these reviews.

## Discussion

This systematic review aimed to evaluate the articles on PA, sport, and exercise behavior change that were published in the first 365 days after WHO declared COVID-19 a pandemic on March 11, 2020. This review is important for the advancement of this research field and its potential to contribute to policy making decisions.

We found that researchers almost all over the world participated by publishing articles in academic journals. Several academic opinion pieces about anticipated negative consequences of the pandemic on PA, exercise, and health were available already before March 11, and these accumulated rapidly thereafter. By May 2020, two dozen empirical studies had been published and many more followed quickly. Subsequently, the first literature review (a scoping review) was published on November 3, 2020 which stated “Most of the evidence identified a decrease in PA levels due to social distancing measures and that PA might help to decrease the mental health burden related to the COVID-19 outbreak” (Caputo and Reichert, 2020, p. 1275). In retrospect, the findings from all four early reviews seem oversimplified, and it would probably have been possible to reach more nuanced conclusions even in the first 365 days after the outbreak of the pandemic.

When we examine the quality of the published original studies, the majority are suboptimal as they are based on convenience samples (with sometimes very small pool of participants) and relied on self-report data (recollection of prepandemic behavior) (Figure 5). There are definitely circumstances where self-report data is useful and might be even more appropriate than device-based measures such as when recording exercise types or exercise preference, etc. (Nigg et al., 2020). However, using questionnaires for PA duration and volume with a small sample size does not seem to be adequate and efficient for either policy-making purposes or informing the research field specifically. In fact, nowadays there are real-world data available through fitness trackers and apps with large sample sizes that can help provide a more accurate picture of PA duration and volume (Ding et al., 2020). In our view, in this research field, there is either a lack of understanding of the optimal fit between measurement methods and constructs to be measured or a lack of resources to conduct more appropriate research. It is important to raise awareness that publication of numerous studies conducted using suboptimal methods, even when study limitations are pointed out in the article, are detrimental to the research field as a whole. Difficulties in methodological and statistical reporting (see Figure 6) present an additional problem.

In the introduction, we pointed out the criticisms that are sometimes targeted at systematic reviews, particularly the criticism that systematic reviews are often conducted mechanistically without addressing the specificities of the research field. Accordingly, we took a more unconventional approach: We have introduced hypotheses about the more trivial expectations of what the pandemic would likely bring in terms of PA, sport and exercise behavior change before retrieving articles and explained some nuances about expected changes that require further examinations. We then employed a very broad literature search string, did not completely disregard the articles that were screened out, and have attempted to capture a maximum of the relevant evidence for describing behavior change that was observed during the pandemic. This method retrieved numerous articles that provided us with an immense diversity of specific research aims and approaches. Consequently, the picture of results in this review is greatly differentiated. This is both in terms of our assessment of research performance and in terms of research findings. This point becomes particularly clear by comparing our main findings with those from the four reviews published in the first 365 days of the pandemic.

We are specifically critical of the fact that many of the original empirical studies only stated the obvious and expected decrease in PA volume, and that this superficial finding was then further highlighted by systematic (or scoping) reviews. The broader approach presented here delineates this general finding considerably. For example, it appears that many individuals in the general population who were already physically active or exercising prior to the pandemic actually managed to remain active during the pandemic (i.e., above the WHO recommended minimum PA volume of 150 MVPA minutes per week). Furthermore, it seems that PA volume dropped significantly in some vulnerable groups (e.g., frail elderly, patients, rehabilitants with severe health conditions) while in some other groups PA did not decrease (e.g., children in Germany) or decreased only slightly (e.g., those who were quite active even before the pandemic). Such specifications would have been extremely helpful for policy makers early in the pandemic. For example, they could have implemented more targeted closures of services and much more tailored preventive actions to avoid sharp decrease in PA in some groups. We assume that there was a high motivation in the field to dramatically emphasize with empirical data mainly any deterioration that occurred because of the pandemic, rather than to paint a more multifaceted picture.

We suggest the following interdependent factors as part of the reasons behind this prevalent type of research on health-related PA, sport and exercise during the pandemic. First, the pressure on researchers to publish (Tiokhin and Derex, 2019; Tiokhin et al., 2021), together with the willingness of some research journals to publish almost anything (Forero et al., 2018) has become preposterous. Second, perhaps the easiest way for researchers to comply with the pressure is to conduct studies that are cheap, easy to organize, and simple to conduct. Third, there may be a reinforcing tendency in the field to maintain conventional approaches (again, mostly because they are so easy to use), even though they have long been criticized. This is particularly evident in the frequent use of the IPAQ (and derived measures) in surveys, which is known to be highly problematic from a methodological point of view (Lee et al., 2011; Kim et al., 2013). Fourth, our analysis reflects how much basic knowledge of physical education, kinesiology, sports, and exercise science have diminished in the field. For example, the positive effects of PA and exercise can only be partially captured through measures of PA volume. Beyond its physiological effects, the mental and social benefits of exercise and sport are largely based on the type of exercise chosen and the setting in which it is performed (Chekroud et al., 2018). It is also surprising that muscle-strengthening activities, which are even emphasized in current WHO recommendations on physical activity, were usually neglected in studies.

## Limitations

In our review, given the large number of articles that were screened and extracted, relatively large numbers of researchers and students were involved. Although intensive training was provided to the team, this may have introduced bias in the article selection process as well as in article coding and data extraction and errors may have occurred. Furthermore, comparing this review with the four previously published reviews discussed above reveals that even the smallest change in the literature search string and the inclusion of different or additional article databases will lead to different search results. However, we are not concerned about having missed a significant number of articles with our search since the list of our retrieved articles is very comprehensive. Finally, our decision to focus only on articles published in the first 365 days after declaration of the pandemic may have contributed to our overall conclusion about the quality of research output being rather negative. It is possible that only these earlier studies were hastily conducted and published, while high quality work appeared later. Even so, this is still problematic since these earlier studies were fundamental in guiding governmental and political decisions at the beginning of the pandemic when the restrictions on PA, sport, and exercise were the most drastic. Although including published data sets in our review alongside the published articles could have helped with our understanding of the nuances in PA, sport, and exercise behavior change, our focus on the quality of published articles arose because we believed that policymakers needed interpreted research to make adequate decisions during the pandemic rather than evaluating data sets themselves.

## Conclusions

This review shows that although there is willingness and commitment among researchers in this field, there is still a lack of structural prerequisites to contribute to high quality research. It is especially important to address these shortcomings in research design and methodology, specifically, given the rising prevalence of skepticism about research and science these days that is being fueled among some in many places around the world.

We further believe that it would be crucial that national stakeholders such as the Centers for Disease Control and Prevention (CDC) in the USA, the UK National Health Service (NHS), Germany's Robert Koch Institute (RKI), and the WHO expand on the existing initiatives such as NHANES or BIObank data sets and implement more longitudinal study cohorts to monitor health parameters, such as PA and exercise, which can be rapidly accessed in the face of sudden changes in the society such as the current pandemic. Until such study cohorts exist, it will be left to chance whether research groups would take the opportunity and contribute with high quality research.

Finally, we believe that research institutions need to be much more explicit about research standards in this field and disseminate information on implementation of high-quality measurements, study designs, and methodology more effectively. Difficulties in the research field need to be pointed out more clearly, as with this review, so that less experienced researchers have a clear benchmark to work with. At the same time, it is paramount that research journals demand higher quality research and refrain from publishing low quality studies. This will help the research on PA, sport, and exercise to grow and contribute more effectively to the management of societal and health crises in the future.

## Data Availability Statement

The original contributions presented in the study are included in the article/Supplementary Materials. Further inquiries can be directed to the corresponding author.

## Author Contributions

This systematic review was initiated by RB. All authors contributed equally to the project and cooperated closely in writing the manuscript. All authors contributed to the article and approved the submitted version.

## Funding

During the analysis and manuscript preparation, ST was supported by the German Academic Scholarship Foundation. Publication of this systematic review was funded by the Deutsche Forschungsgemeinschaft (DFG, German Research Foundation)—Project number 491466077. This funding body had no role in the study design, analysis, interpretation of findings, or writing of the manuscript.

## Conflict of Interest

The authors declare that the research was conducted in the absence of any commercial or financial relationships that could be construed as a potential conflict of interest.

## Publisher's Note

All claims expressed in this article are solely those of the authors and do not necessarily represent those of their affiliated organizations, or those of the publisher, the editors and the reviewers. Any product that may be evaluated in this article, or claim that may be made by its manufacturer, is not guaranteed or endorsed by the publisher.
