# Supplementary material for: Using COVID-19 Pandemic as a Prism: A Systematic Review of Methodological Approaches and the Quality of Empirical Studies on Physical Activity Behavior Change
Source: Front Sports Act Living. 2022 Apr 21;4:864468. doi: 10.3389/fspor.2022.864468 (PMC9069113; doi:10.3389/fspor.2022.864468)
Supplement: Supplementary file 1 [file Table_1.DOCX]

# ADMINISTRATIVE INFORMATION

## Date: July 2, 2021

##

## Title

**Identification**

COVID-19 and PA behavior change methodology review: a systematic review protocol

**Update**

This protocol is not for an update of a previous systematic review.

## Registration

This systematic review was not registered.

## Authors

**Contact**

Ralf Brand, Sinika Timme, Constantin Späth
Sport and Exercise Psychology, University of Potsdam, Potsdam, Germany
Department of Kinesiology, Iowa State University, Ames, IA, United States
ralf.brand@uni-potsdam.de, stimme@uni-potsdam.de, spaeth@uni-potsdam.de

Campus I:
Am Neuen Palais 10
14469 Potsdam
Location: 1.12.1.16b

Sanaz Nosrat
Department of Health Sciences, Lehman College/CUNY, New York, NY, United States
[SANAZ.NOSRAT@LEHMAN.CUNY.EDU](mailto:SANAZ.NOSRAT@LEHMAN.CUNY.EDU)

250 Bedford Park Blv West,

Bronx, NY, 10468

**Contributions**

The guarantor of the review is Ralf Brand.

## Amendments

This protocol is the revised version of an originally less elaborated one. The initial aim of this systematic review was to create a more differentiated review in regards to the changes in physical activity (PA), sport, and exercise behavior induced by the COVID-9 pandemic and to test whether the mainly unidirectional conclusions of a simple decrease in PA published by other systematic reviews can be supported. In our endeavor to overcome the mechanistic approach of standard systematic reviews which are not suitable for the vast majority of scientific topics and their inherent complexities, we discovered the hermeneutic approach published by Boell & Cecez-Kecmanovic (2014). Because of that discovery and the fact that we were faced (as we assumed) with a huge heterogeneity in the data we had already screened, we decided to change our aim. Our new goal was to explore and evaluate the performance and state of research on PA behavior change during COVID-19-related early governmental restrictions in the first 365 days after the pandemic was declared.

## Support

**Sources**

There is no external funding to conduct this review.

**Sponsor**

*NA*

**Role of sponsor/funder**

*NA*

# INTRODUCTION

## Rationale

The COVID-19 pandemic induced a massive amount of change in people´s lives worldwide. Governments had to install non-pharmaceutical interventions (NPIs) to mitigate the spread of SARS-CoV-2. It is beyond question that these imposed NPIs have affected many aspects of people´s lives, including PA, sport, and exercise. PA has been implicated in the management and prevention of many chronic disease conditions and early in the pandemic, published data indicated that PA could also potentially reduce morbidity and mortality of viral infection with SARS-CoV-2. Therefore, behavioral scientists called for action suggesting an international PA and public health research agenda to inform policies and practices.

## Objectives

Our aim is to review the performance of research field on PA, sport, and exercise and how they responded to the urge for more research during the early days of the COVID-19 pandemic.

# METHODS

## Eligibility criteria

We will include:

(1) Any empirical research article contributing direct evidence about changes in PA, sport, or exercise behavior during COVID-19 compared to pre COVID-19 in the first year after the declaration of the pandemic by WHO, and

(2) and published in English or German.

## Information sources

We will search the Social Sciences Citation index, the Emerging Sources Citation Index, Medline, PubMed Central (PMC) and all other sources included in the Web of Science platform and the Pubmed database.

## Search strategy

The search string [("Physical activit*" OR "Exercis*" OR "Sport*") AND ("COVID*" OR "SARS-CoV-19")] will be used. Additionally, the search will be restricted by the filters "Human", "English", "German". Publications up to March 11, 2021 will be considered.

## Study records

**Data management**

We will use the Covidence software to manage records and data throughout the review.

**Selection process**

In each phase of the review at least two independent reviewers will be involved. Conflicts will be solved with the main authors.

**Data collection process**

We will extract data from reports in Covidence using a self-created “Data Extraction Template” as well as a “Quality Assessment Template”. These are based on criteria from the JBI checklists for the analytical cross-sectional and the prevalence studies, and the AXIS appraisal tool for the cross-sectional studies. Two independent reviewers will extract information separately. Conflicts will be resolved by Ralf Brand.

## Data items

For all articles, the standard bibliographic information (e.g., title and abstract of the article, year of publication, and journal title), the nationality of the first author via his professional affiliation, and the exact publication date will be extracted. In addition, methodologically relevant information will be extracted. This includes characteristics of the sample (e.g., nationality, and study population), study design (e.g., duration and type of sampling relative to the stringency of the health containment measures in the respective country, when possible; number of measurement time points), measurement method (e.g., device-based measurement, self-report), and measurement content (e.g., names of questionnaires used).

## Outcomes and prioritization

*NA*

## Risk of bias in individual studies

The quality of methodology will be assessed by items based on criteria from the JBI checklists for the analytical cross-sectional and the prevalence studies, and the AXIS appraisal tool for cross-sectional studies:

1. Are the aims/objectives of the study clearly stated?
2. Are the study participants described in detail?
3. Are the measured outcomes (related to PA) clearly defined?
4. Are the statistical results adequately documented?
5. Are the methods adequately documented?

## Data Synthesis

Because of the heterogenous nature of the publications, no quantitative synthesis beyond frequency counts will be performed.

## Meta-bias(es)

*NA*

## Confidence in cumulative evidence

*NA*
